# Prenatal Diagnosis of Sex Chromosome Aneuploidies: A Retrospective Study Using QF-PCR, SNP-Based Chromosomal Microarray Analysis, and NIPT

**DOI:** 10.3390/genes17020171

**Published:** 2026-01-31

**Authors:** Irina Ioana Iordanescu, Andreea Catana, Zina Barabas Cuzmici, Paula Chelu, Bianca Florentina Basangiu, Emilia Severin, Mariela Sanda Militaru

**Affiliations:** 1 Genetics Department, “Carol Davila” University of Medicine and Pharmacy, 020027 Bucharest, Romania; irina-ioana.iordanescu@drd.umfcd.ro; 2 Genetics Department Laboratory, Regina Maria, 060044 Bucharest, Romania; andreea.catana@reginamaria.ro (A.C.); barabaszina@yahoo.com (Z.B.C.); paula.chelu@reginamaria.ro (P.C.); florentina.basangiu@reginamaria.ro (B.F.B.); mariela.militaru@reginamaria.ro (M.S.M.); 3 Genetics Department, “Iuliu Hațieganu” University of Medicine and Pharmacy, 400347 Cluj-Napoca, Romania

**Keywords:** sex-chromosome, aneuploidy, prenatal diagnosis, QF-PCR, SNP-array, NIPT

## Abstract

**Objectives:** This study aimed to characterize the types and frequencies of sex chromosome aneuploidies (SCAs) detected through invasive prenatal testing, evaluate the concordance between non-invasive prenatal testing (NIPT) and confirmatory diagnostic methods, and assess the challenges faced during genetic counseling following SCA diagnosis. **Study Design:** A retrospective review was conducted on 842 prenatal samples collected between 2020 and 2024 in a tertiary private medical center. Samples included amniotic fluid, chorionic villi, and products of conception. Testing involved rapid QF-PCR for aneuploidy detection, followed by SNP-based chromosomal microarray analysis (CMA). NIPT results with high risk for sex chromosomes aneuploidies were correlated with invasive testing outcomes in 19 cases. **Results:** Sex chromosome aneuploidies were identified in 67 cases (7.96%), with Turner syndrome (45, X) being the most frequent (23 cases, including six mosaics), followed by Klinefelter syndrome (18 cases), 47, XYY (14 cases), and trisomy X (12 cases). Among 19 NIPT-tested cases, 10 were true positives, 5 false positives, and 4 false negatives, including two mosaic Turner syndrome cases undetected by NIPT. Discordances were attributed to factors such as mosaicism and placental anomalies. **Conclusions:** Prenatal diagnosis of SCAs via invasive testing remains crucial due to NIPT’s limited sensitivity for mosaicism and false positives. Comprehensive genetic counseling is essential to navigate diagnostic uncertainties and optimize prenatal management and postnatal outcomes.

## 1. Introduction

Sex chromosome aneuploidies (SCAs) are among the most common chromosomal abnormalities, with an estimated prevalence of 1 in 400 live births [1]. These conditions are highly heterogeneous, both in phenotype and clinical severity, and their prognosis often depends on a combination of prenatal ultrasound findings and the quality of postnatal follow-up. This complexity makes the communication of diagnostic results following amniocentesis or chorionic villus sampling (CVS) particularly challenging during post-test genetic counseling [2]. It is estimated that 75–90% of individuals with SCA remain undiagnosed throughout life or are diagnosed later, often in the context of infertility [3]. Studies suggest that early hormonal therapy may have beneficial effects on neurobehavioral outcomes [4]. Furthermore, early detection through prenatal screening enables timely management and may significantly improve quality of life in affected individuals [5].

Over a four-year period (2020–2024), our clinic processed 842 single nucleotide polymorphism (SNP) array tests derived from ongoing pregnancies or products of conception. Out of these, 28 cases presented with sex chromosome abnormalities: 11 from amniotic fluid samples, 5 from chorionic villi, and 12 from products of conception. These 28 cases represent a subset of sex chromosome abnormalities identified through SNP-array analysis, whereas the complete cohort of prenatally diagnosed sex chromosome aneuploidies, including cases detected by QF-PCR, karyotyping, and FISH, comprised 67 cases, as detailed in the Results Section. All cases identified in products of conception involved monosomy X (Turner syndrome), with miscarriages occurring during the first trimester. Turner syndrome is associated with a high rate of spontaneous fetal loss between 10 weeks of gestation and term, with estimates ranging between 80 and 95% [6].

Among ongoing pregnancies, we diagnosed six cases of monosomy X. One of these showed a mosaic pattern with under 20% of cells containing a Y chromosome; another presented with a 23 Mb copy number variant (CNV) on the short arm of the X chromosome. Additionally, three cases of trisomy X, two of Klinefelter syndrome (47, XXY), and one rare case of 48, XXYY were identified. Most patients had undergone non-invasive prenatal testing (NIPT) prior to invasive procedures, motivated by either a high-risk result for SCA or abnormal ultrasound findings such as increased nuchal translucency or, in one case, diaphragmatic hernia.

A total of 19 NIPT tests in our cohort raised concern for SCA: 13 had inconclusive results or insufficient DNA (four cases), all of which were followed by normal results on confirmatory invasive testing. Of the remaining six, one case with high risk for Klinefelter syndrome was confirmed, while among five cases with high risk for Turner syndrome, three were not confirmed. These findings are consistent with the current literature indicating that the detection rate and positive predictive value (PPV) of NIPT for SCAs are lower compared to autosomal trisomies. False positives are especially frequent in monosomy X and may be explained by factors such as maternal mosaicism, maternal trisomy X, placental mosaicism, or GC-content bias during sequencing [7]. However, variability in positive predictive value has been reported between conditions and testing methodologies, emphasizing that positive NIPT results require confirmation by invasive diagnostic procedures [8].

Monosomy X (Turner syndrome) is the most frequently detected SCA mosaicism in prenatal diagnosis. Diagnostic tools include karyotyping, chromosomal microarray analysis (CMA), and fluorescence in situ hybridization (FISH), which are complementary and often necessary in combination for accurate diagnosis [9,10]. Turner syndrome is characterized by complete or partial absence of one X chromosome in females, commonly caused by meiotic nondisjunction. Besides the classic 45, X karyotype, Turner syndrome may present in mosaic forms such as 45, X/46, XX or 45, X/46, XY. The presence of Y chromosome material increases the risk of gonadoblastoma and warrants surgical evaluation [11,12,13].

Structural variants of the X chromosome can also be seen in Turner syndrome, including isochromosomes (46, X,i(Xq), 15–18%), ring chromosomes (46, X,r(X), ~6%), and deletions involving either the short (Xp) or long arm (Xq) [14]. Genotype–phenotype correlations remain partially understood. For example, patients with 45, X/46, XX mosaicism tend to have fewer comorbidities, while those with ring chromosomes often display more severe phenotypes [15].

Klinefelter syndrome (47, XXY) is the most frequent SCA in males, with a prevalence of 1 in 660 live births [16]. Although clinical features such as developmental delay, learning difficulties, and infertility may be present from childhood, the diagnosis is often delayed or missed. It is estimated that 65–76% of cases remain undiagnosed, with only 6% detected in childhood and 9% in adulthood [17]. Prenatal diagnosis accounts for approximately 10% of identified cases, largely due to increased use of NIPT [18,19,20].

A rarer form of SCA is 48, XXYY syndrome, typically of paternal origin due to postzygotic mitotic nondisjunction. Affected individuals often display behavioral challenges such as impulsivity, anxiety, social difficulties, and attention-deficit/hyperactivity disorder (ADHD). Neuroimaging studies have shown smaller overall brain volume, with white matter abnormalities and reversed gray/white matter patterns in key brain regions linked to behavior [21,22].

First-trimester combined screening, which includes serum markers (free β-hCG and PAPP-A) and ultrasound measurements (nuchal translucency and nasal bone) performed between 11 and 13 weeks of gestation, has a reported detection rate of 79–90% for trisomy 21, with a false positive rate around 5% [23]. Although initially developed for autosomal trisomies, this screening approach can also detect SCAs. However, the advent of NIPT has shifted the paradigm, as it offers superior sensitivity for trisomy 21 and is increasingly used as a first-line screening tool. Its performance for SCAs, while promising, remains limited by lower predictive values and a higher rate of false positives [18].

Our study aims to provide insights into the clinical and counseling implications of prenatal detection of sex chromosome aneuploidies (SCAs) through a four-year retrospective review of cases analyzed at our center. The specific objectives are as follows: (1) to characterize the types and frequencies of SCAs detected via chorionic villus sampling, amniocentesis, and analysis of products of conception; (2) to assess the diagnostic concordance between non-invasive prenatal testing (NIPT) and confirmatory invasive testing; and (3) to examine the genetic counseling challenges encountered during post-test communication and follow-up in cases involving SCAs.

## 2. Materials and Methods

Between January 2020 and December 2024, our multidisciplinary prenatal genetic team evaluated 842 cases referred for invasive prenatal testing, performing the study in a tertiary private medical center. Indications for testing included abnormal first-trimester ultrasound findings (e.g., increased nuchal translucency, diaphragmatic hernia), high-risk results from non-invasive prenatal testing (NIPT), or previous pregnancy history suggestive of chromosomal abnormalities. Specimens consisted of amniotic fluid, chorionic villi, or products of conception (POC) following miscarriage. All samples were processed using a two-step approach: rapid aneuploidy detection via QF-PCR, followed by chromosomal microarray analysis (CMA) using SNP-based technology.

QF-PCR was performed using the Devyser v3 Compact kit (Devyser AB, Stockholm, Sweden), targeting 26 STR markers located on chromosomes 13, 18, 21, X, and Y. The reaction products were analyzed on the ABI 3500 Genetic Analyzer (Applied Biosystems, Waltham, MA, USA), and allelic profiles were interpreted with GeneMapper® ID-X software, version 1.5 (Applied Biosystems).This assay served both to confirm numerical abnormalities of the sex chromosomes and to rule out maternal cell contamination, particularly in POC samples.

Chromosomal microarray analysis (CMA) was conducted using the Affymetrix CytoScan 750K platform (Thermo Fisher Scientific, Santa Clara, CA, USA), which provides a resolution of approximately 100 kb.This hybrid array contains ~750,000 markers for copy number and 200,000 SNP probes, enabling the detection of copy number variants (CNVs), regions of homozygosity, and mosaicism above 15–20%. Data analysis was carried out using Chromosome Analysis Suite (ChAS) software, version 4.3 (Thermo Fisher Scientific), aligned to GRCh38/hg38 and cross-referenced with public databases including OMIM, ClinVar, DECIPHER, and NCBI. Balanced rearrangements, point mutations, and low-level mosaicism below the detection threshold of CMA were not evaluated by this method.

A total of 19 patients in this cohort underwent NIPT prior to invasive sampling due to suspicion of sex chromosome aneuploidies (SCAs). Correlation between NIPT results and confirmed diagnoses was assessed, noting both true positive and false positive outcomes. Cases in which NIPT suggested SCA but invasive testing failed to confirm the finding were further evaluated considering potential biological explanations such as confined placental mosaicism or maternal sex chromosome anomalies. Two mosaic Turner syndrome cases with normal ultrasound examinations and false-negative NIPT results are discussed in relation to the previous literature (e.g., [Cytogenetics and Molecular Investigations detect a Mosaic Variant of Turner Syndrome only Suspected by Non-Invasive Prenatal Testing: Two Case Reports with Negative Ultrasound Examinations]).

## 3. Results

Out of the 842 prenatal samples analyzed between 2020 and 2024, 67 cases (7.96%) were diagnosed with sex chromosome aneuploidies (SCAs) through invasive testing, including QF-PCR, conventional karyotyping, FISH, and SNP-array analysis.

These included the following:

- 45, X (Turner syndrome): 23 cases (including 6 mosaics);
- 47, XXY (Klinefelter syndrome): 18 cases;
- 47, XYY: 14 cases;
- 47, XXX: 12 cases.

Among the 23 Turner syndrome cases, 6 (26.1%) were identified as mosaic, with variable proportions of the 45, X cell line detected by SNP array. The remaining 17 presented with a pure 45, X profile.

Of the 67 cases, 19 patients had undergone non-invasive prenatal testing (NIPT) prior to referral, specifically for suspected SCAs. Among these,

- A total of 10 NIPT results were concordant with the invasive diagnosis (true positives), including three 47, XXY, two 47, XYY, two 47, XXX, and three 45, X.
- Five cases were false positives, where NIPT suggested an SCA that was not confirmed by invasive testing. In three of these, maternal chromosomal polymorphisms or confined placental mosaicism were considered as possible causes of discordance.
- Four cases were false negatives, with normal NIPT results despite an SCA being identified invasively. Notably, two of these were mosaic 45, X/46, XX Turner syndrome, undetected by NIPT due to low-level mosaicism, below the detection threshold of the technique.

These two mosaic Turner syndrome cases, both with normal first-trimester ultrasound findings, reinforce concerns about the sensitivity of NIPT in detecting low-grade mosaicism and support findings reported in the previous literature. In both instances, QF-PCR confirmed the presence of an abnormal sex chromosome complement, and CMA revealed mosaicism with 45, X cell lines present in 30–40% of cells.

A full breakdown of SCA types, NIPT concordance, and mosaic proportions is presented in Table 1.

Among these cases, several presented with complex or discordant results between screening and confirmatory diagnostic testing, particularly involving sex chromosome abnormalities and mosaicism. The following four cases are illustrative examples that highlight diagnostic challenges and the utility of combined cytogenetic and molecular approaches in prenatal diagnosis.

### 3.1. Case 1

**Figure 1:**
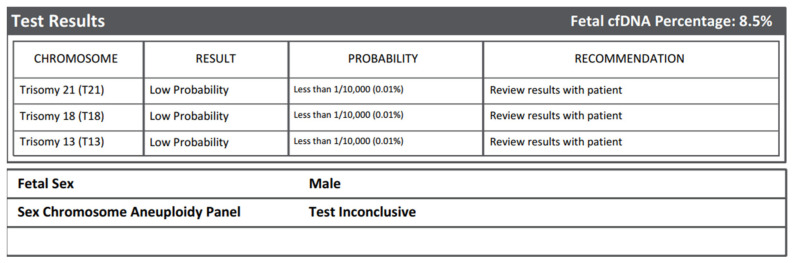
Non-invasive prenatal testing (NIPT) report indicating male fetal sex prediction and an inconclusive result for sex chromosome aneuploidies (SCAs). This result is consistent with the information summarized for Case 1 in Table 2 and prompted further invasive diagnostic investigations.

A 28-year-old pregnant woman underwent non-invasive prenatal testing (NIPT) as part of routine first-trimester screening. The test reported a low risk for trisomy 13, 18, and 21, and predicted male fetal sex, but yielded an inconclusive result for sex chromosome aneuploidies (SCA) (Figure 1). First-trimester ultrasound examination was normal, and notably, female fetal anatomy was observed, in discordance with the NIPT-reported sex.

**Table 2:** Summary of four cases with suspected or confirmed sex chromosome aneuploidy.

Case	Maternal Age (Years)	NIPT Result	Ultrasound Findings	QF-PCR	Karyotype	SNP Array	Final Diagnosis
1	28	Male sex; inconclusive for SCAs	Normal; female fetal sex	Negative for autosomal aneuploidies; no SRY; suggestive of 45, X	45, X	Monosomy X with low-level Y material (mosaicism < 20%)	Mosaic Turner syndrome with Y material
2	31	High risk for monosomy X	Normal	T1 and T3 markers with 1:1 ratio; others noninformative	45, X[43]/46, X+mar[8]	Large deletions on Xp (35.3 Mb) and Xq (73.6 Mb); marker of X origin	Mosaic Turner syndrome with X marker
3	39	High risk for monosomy X	Not specified	Inconclusive (×2 labs); negative for autosomal aneuploidies	46,X,i(X)(q10)[17]/45,X[3]	Loss of Xp, gain of Xq → consistent with isochromosome Xq	Mosaic Turner with i(Xq)
4	25	Not specified (indication: NT ↑)	Increased NT	Not performed (CVS used for SNP array)	Not performed	Two X and two Y chromosomes (48, XXYY); no pathogenic CNVs reported	48, XXYY syndrome

↑ indicates increased nuchal translucency (NT).

**Figure 2:**
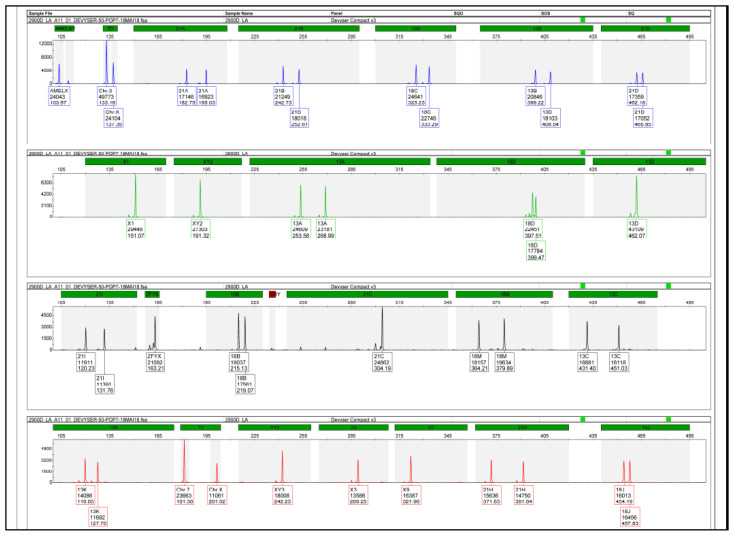
QF-PCR profile obtained from amniotic fluid showing a Turner syndrome-compatible pattern. Absence of the SRY marker and monoallelic amplification of X-chromosome STR markers are observed, consistent with monosomy X (45, X).

QF-PCR analysis performed on amniotic fluid excluded common autosomal trisomies and showed no amplification of the SRY gene, with an STR profile consistent with Turner syndrome (45, X) (Figure 2). Given the initial male sex prediction by NIPT, further investigations were pursued to clarify the discrepancy.

**Figure 3:**
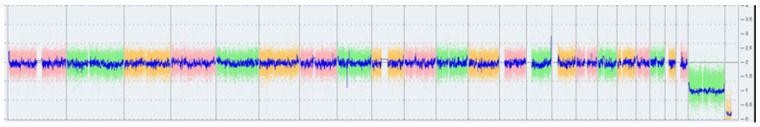
SNP-array profile showing monosomy X with low-level Y chromosome signal.

Conventional karyotyping revealed a 45, X karyotype, consistent with Turner syndrome. To further explore the discrepancy, SNP-array analysis was performed. The array confirmed monosomy X but also revealed a non-zero Y chromosome ratio below the mosaicism detection threshold of 20% (Figure 3), indicating the possible presence of a low-level Y cell line.

These findings raised suspicion for low-grade mosaicism: 45, X/46, XY, not detected by karyotype but partially reflected in the SNP-array data. The mother’s karyotype (46, XX) was normal, ruling out maternal cell contamination.

The case was referred for genetic counseling, and gonadectomy was discussed as a postnatal preventive measure due to the risk of gonadoblastoma associated with Y chromosome material in Turner mosaicism.

### 3.2. Case 2

A 31-year-old pregnant patient underwent non-invasive prenatal testing (NIPT) as part of routine first-trimester screening. The NIPT report indicated a high risk for monosomy X, suggestive of Turner syndrome. Following genetic counseling, invasive prenatal testing was carried out at 16 weeks of gestation.

**Figure 4:**
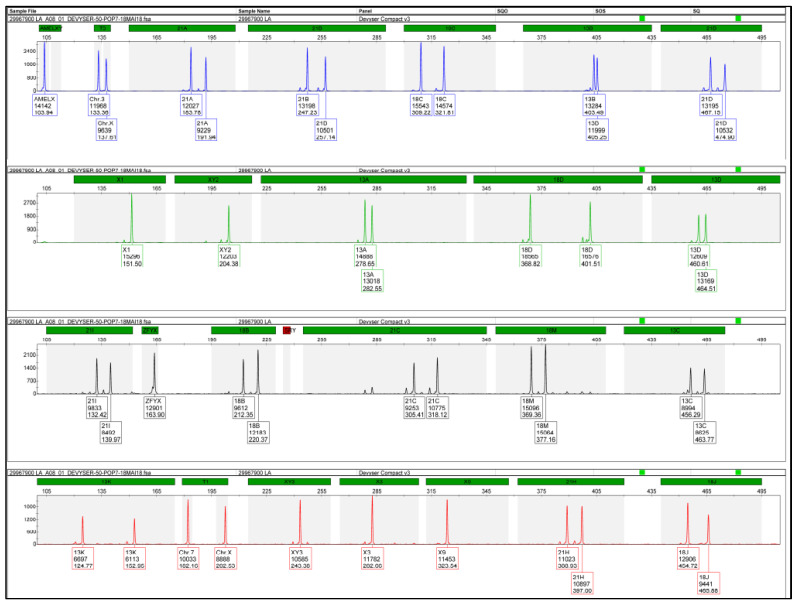
QF-PCR profile showing partial informativeness for X-chromosome STR markers. Two non-polymorphic markers (T1 and T3) display a 1:1 allelic ratio, while the remaining markers are non-informative, prompting further cytogenetic and SNP-array analysis.

Quantitative fluorescent PCR (QF-PCR) analysis of amniotic fluid excluded autosomal aneuploidies. Regarding the sex chromosomes, two non-polymorphic STR markers (T1 and T3) displayed a 1:1 ratio, while the remaining eight markers were uninformative. Although the assay indicated female fetal sex, the partially informative STR profile prompted further investigation (Figure 4).

**Figure 5:**
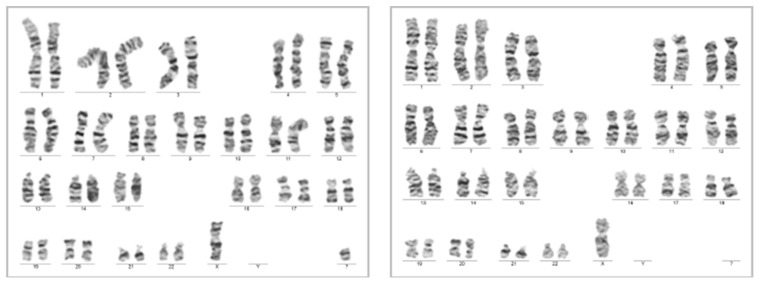
Karyotype with mosaicism: 45, X[43]/46, X+mar[8].

Subsequent conventional karyotyping identified chromosomal mosaicism: 45, X in 43 metaphases and 46, X+mar in eight metaphases, 45, X[43]/46, X+mar[8], suggesting a predominant monosomy X cell line with a subpopulation harboring an additional marker chromosome (Figure 5). The origin of the marker chromosome could not be determined cytogenetically, warranting higher-resolution analysis.

To elucidate the nature of this marker, SNP-array analysis was performed. The array identified the marker chromosome as derived from the X chromosome. Specifically, the SNP-array detected two large copy number losses:

- A 35.3 Mb deletion on the short arm of chromosome X (Xp22.33–p21.1);
- A 73.6 Mb deletion on the long arm (Xq21.1–q28).

**Figure 6:**
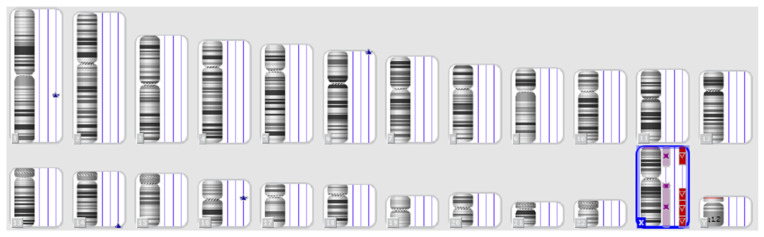
SNP-array result showing large CNVs on both arms of the X chromosome.

These copy number variations (CNVs) explained the partial informativeness observed in the QF-PCR and confirmed that the marker chromosome represented structurally abnormal X chromosomal material (Figure 6).

Based on these findings, the case was classified as mosaic Turner syndrome with a structurally altered second X chromosome. The family was counseled regarding the possible phenotypic variability and implications of this mosaic karyotype.

### 3.3. Case 3

A 39-year-old pregnant patient underwent NIPT screening, which revealed a high risk for monosomy X. Following genetic counseling, the patient expressed her intention to continue the pregnancy regardless of the findings and opted for a comprehensive diagnostic evaluation.

QF-PCR analysis was performed in two independent laboratories, with both results inconclusive for sex chromosome aneuploidies and negative for autosomal aneuploidies.

To further clarify the diagnosis, FISH analysis was performed using a centromeric probe for the X chromosome (DXZ1) on 200 interphase nuclei. The test identified two distinct cell lines:

- Total of 90% of cells (180/200): two centromeric signals from chromosome X (normal female complement);
- Total of 10% of cells (20/200): a single centromeric signal from chromosome X (monosomy X).

→ FISH result: nuc ish (DXZ1×1) [20/200], indicating mosaic monosomy X, consistent with Turner syndrome.

Karyotyping confirmed this mosaicism with the following formula, 46,X,i(X)(q10)[17]/45,X[3], indicating the following:

- A major cell line (85%) with an isochromosome of the long arm of the X (i(Xq))—resulting in a duplication of Xq and deletion of Xp;
- A minor cell line (15%) with monosomy X;

SNP-array analysis supported these findings, revealing the following:

- A loss of the short arm (Xp);
- A gain of the long arm (Xq).

→ consistent with the presence of an isochromosome Xq in mosaicism.

**Figure 7:**
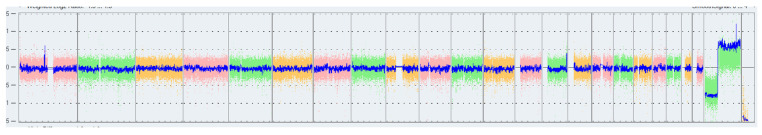
SNP-array result showing Xp loss and Xq gain, confirming the presence of isochromosome Xq.

These copy number variations are consistent with the presence of an isochromosome Xq in mosaic form (Figure 7).

### 3.4. Case 4

A 25-year-old patient was referred for prenatal genetic testing due to increased nuchal translucency noted on first-trimester ultrasound. Chorionic villus sampling (CVS) was performed, and SNP-array analysis revealed the presence of the following:

- Two X chromosomes;
- Two Y chromosomes.

**Figure 8:**
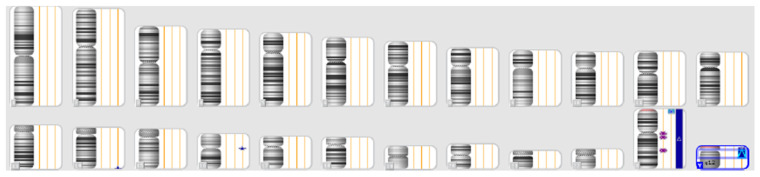
SNP-array result confirming two X and two Y chromosomes.

This result is consistent with a rare sex chromosome aneuploidy: 48, XXYY (Figure 8).

No other significant copy number changes were identified. Genetic counseling was offered regarding the implications of this condition, including potential developmental, cognitive, and behavioral outcomes associated with 48, XXYY syndrome.

A summary of the four cases with suspected or confirmed sex chromosome aneuploidies is presented in Table 2, including maternal age, NIPT results, ultrasound findings, QF-PCR outcomes, karyotype, SNP-array data, and final diagnosis.

## 4. Discussion

This four-year series adds to growing evidence that non-invasive prenatal testing (NIPT), while highly reliable for common autosomal trisomies, has limited positive predictive value (PPV) for sex chromosome aneuploidies (SCAs), particularly monosomy X [24,25].

### 4.1. NIPT Performance in SCAs

The discussion of NIPT performance in this section is based primarily on previously published studies and is intended to provide clinical context for interpreting discordant cases observed in our cohort, rather than to represent a formal performance evaluation within the study population.

Our cohort revealed that one true positive (47, XXY) and three false-positive monosomy X findings align with the literature reporting a PPV of 38–50% for SCAs, with approximately 21% PPV for 45, X, but markedly higher values for other sex chromosome trisomies (e.g., 52.9% for 47, XYY) [24,25]. A large retrospective study (n = 222,107) reported an overall SCA PPV of 45.4%, with a monosomy X PPV of approximately 17.5%, underscoring the unreliability of NIPT as a standalone diagnostic tool for 45, X. Although genome-wide NIPT platforms have demonstrated the potential to detect atypical chromosomal abnormalities and selected copy number variations beyond common trisomies, the clinical interpretation of such findings remains challenging and necessitates confirmatory invasive testing [26,27].

### 4.2. Causes of Discordant Results

Several biological and technical factors contribute to false-positive NIPT results for SCAs, including maternal mosaicism or age-related X-chromosome loss, confined placental mosaicism, and GC-content bias affecting sex chromosomes [27]. Our Case 1, with inconclusive NIPT sex findings but mosaic Turner syndrome with Y chromosome material, and Case 2, presenting complex marker-derived mosaicism, illustrate how cytogenetic and molecular follow-up testing is essential for accurate prenatal diagnosis. In line with previous reports, chromosomal mosaicism frequently leads to inconsistent results across different diagnostic techniques, and the integrated use of conventional karyotyping together with high-resolution copy number-based methods and rapid aneuploidy assays such as QF-PCR significantly improves diagnostic yield in high-risk pregnancies [28].

### 4.3. Mosaicism and Structural Variants

The detection limits of both NIPT and QF-PCR become particularly evident in mosaic or structurally abnormal SCAs. Case 3 illustrates how isochromosome Xq mosaicism escaped NIPT detection and was identified only through FISH, karyotyping, and SNP-array analysis, consistent with previous reports in the literature [29]. Also consistent with previous reports, chromosomal mosaicism frequently leads to discordant findings across different detection methods, and the combined use of karyotyping, chromosomal microarray analysis, and FISH substantially improves diagnostic accuracy in the prenatal setting [30].

### 4.4. Clinical Implications and Counseling

Given that invasive testing remains the gold standard for prenatal diagnosis of SCAs, positive or inconclusive NIPT results must be interpreted cautiously. Professional guidelines recommend confirmatory invasive testing whenever SCAs are suspected, particularly in the presence of abnormal ultrasound findings such as increased nuchal translucency [31]. In our series, Case 4 highlights the value of definitive diagnostic procedures even when ultrasound findings are limited, as SNP-array analysis revealed a 48, XXYY karyotype in the absence of overt sonographic anomalies.

### 4.5. Limitations of the Study

This retrospective, single-center study may not capture the full spectrum of population variability or detect very rare structural chromosomal abnormalities. The relatively small number of cases with available NIPT results (n = 19) limits the ability to perform formal performance validation of NIPT. Rather, the NIPT data were analyzed descriptively, in a real-world clinical context, to illustrate concordance and discordance patterns between screening results and confirmatory invasive diagnostic methods. Additionally, long-term follow-up data were unavailable for some ongoing pregnancies. Despite these limitations, our findings underscore the essential role of integrated genetic diagnostic approaches—combining QF-PCR, karyotyping, FISH, and SNP-array analysis—in resolving ambiguous NIPT findings and supporting accurate prenatal counseling and clinical decision-making.

## 5. Conclusions

This study highlights the diagnostic challenges associated with prenatal detection of sex chromosome aneuploidies (SCAs) and confirms that, while non-invasive prenatal testing is a valuable screening tool, its performance for SCAs—particularly monosomy X—remains limited. Our findings emphasize that confirmatory invasive testing, using an integrated approach combining QF-PCR, conventional karyotyping, FISH, and SNP-array analysis, is essential for accurate prenatal diagnosis. Given the marked phenotypic variability and frequent absence of specific ultrasound findings, especially in mosaic cases, comprehensive and individualized genetic counseling remains crucial to support informed decision-making.

## Figures and Tables

**Table 1:** Summary of sex chromosome aneuploidies (SCA) diagnosed by genetic analysis of samples obtained via invasive procedures, and correlation with NIPT results (n = 67).

SCA Type	Total Cases (n)	Mosaic Cases (n)	NIPT Performed (n)	True Positives	False Positives	False Negatives
45, X (Turner)	23	6	7	3	1	2
47, XXY (Klinefelter)	18	0	4	3	1	0
47, XYY	14	0	3	2	1	0
47, XXX	12	0	5	2	2	0
Total	67	6	19	10	5	2

## Data Availability

The data that support the findings of this study are not openly available due to reasons of sensitivity and are available from the corresponding author upon reasonable request. Data are in controlled access data storage at Regina Maria Hospital—Bucharest.
